# Epidemiological Pattern of Musculoskeletal Injuries in Children Aged 16 Years and Below in a Regional Trauma Centre in Nigeria

**DOI:** 10.7759/cureus.38125

**Published:** 2023-04-25

**Authors:** David O Odatuwa-Omagbemi, Emeka Izuagba, Roy E Enemudo, Cletus I Otene, Nnamdi C Ijezie

**Affiliations:** 1 Surgery/Orthopaedics, Delta State University, Abraka, NGA; 2 Orthopaedics and Traumatology, National Orthopaedic Hospital, Lagos, NGA; 3 Surgery/Plastic Surgery, Delta State University, Abraka, NGA

**Keywords:** traffic accidents, falls, injuries, musculoskeletal, children

## Abstract

Introduction

Injuries in children and adults contribute a large percentage to the global burden of disease. Findings in this study will help authorities and governments in our clime to make policies aimed at the prevention and reduction of this burden.

Methods

This study is a retrospective review of cases of musculoskeletal injuries in children aged 0-16 years seen at the National Orthopaedic Hospital, Lagos, Nigeria, over a period of three years (from January 2017 to December 2019).

Results

Ninety children were included in this study, made up of 58 males (64.4%) and 32 females (35.4%), presenting a male: female ratio of 1.8:1. The combined average age of the children of both sexes was 8.15+/-4.03 years. The home was the most common place (47.8%) where injuries took place, followed by streets/roads (25.6%). Fall was the commonest etiology/mode of injury (57.8%), followed by traffic accidents (23.3%). The 90 patients studied had 96 injuries, of which 92 (95.8%) were close injuries, and the rest were open injuries. The children sustained 101 fractures of individual bones; the femur was the most frequently fractured bone (36, 35.6%), followed by the humerus (30, 29.7%). Treatment modalities offered included closed reduction with casting, open/closed reduction and K-wire fixation of fractures, wound debridement/care for open injuries, and others.

Conclusion

Falls and traffic accidents were responsible for most of the injuries in the children studied. Appropriate policies by those in government/authority and the right measures by parents and caregivers will help to reduce the incidence of these largely preventable injuries.

## Introduction

It was estimated that in 2013, 973 million people sustained injuries that warranted some type of health care, and 48 million people died from injuries globally [1]. Trauma is globally a major cause of morbidity and mortality in children and young persons [1-8]. In the United States (US), over 10 million children are treated for injuries each year, with over 9,000 deaths. Trauma accounts for about 50% of deaths in children after the first year of life in the US [3,5,9].

In developing countries, trauma is the third leading cause of death after infectious diseases and diarrhea, especially in children under five [5,10]. Regional variations in the incidence and etiological factors for injuries in children have been demonstrated in various studies with low-income countries reported as contributing a larger chunk of the global burden of musculoskeletal injuries in children [1,2,11,12].

Musculoskeletal injuries include injuries to skeletal tissues causing fractures, dislocations, and injuries to muscle units, tendons, ligaments, and surrounding soft tissues [5,13].

Etiological factors for injuries in children include but are not limited to falls, traffic accidents, sporting activities, non-accidental injuries, and others, with most of the injuries occurring either at home, in the street, at school, and during sports, or on recreational grounds [5,6,11,14,15]. Commonly observed injuries in the pediatric age group usually range from fractures to dislocations, sprains, ligament injuries, lacerations, etc. [5-7,13].

A child is not a small adult, hence lacks the understanding of how to protect itself from environmental hazards, and as such, must be protected by putting the right measures in place. The outcome of this study, aside from contributing to the general body of scientific knowledge, will help policymakers to formulate the appropriate policies and decide on the type of advocacy and public enlightenment measures that are aimed at preventing and thus reducing the incidence of childhood injuries in our country. This will, in turn, help parents to seek early care at appropriate places when their children are injured and avoid delays, which commonly lead to complications and poorer outcomes.

## Materials and methods

This study involved the review of cases of musculoskeletal injuries in children between the ages of 0-16 years over a period of three years (from January 2017 to December 2019) at the National Orthopaedic Hospital, Lagos, Nigeria.

Data were gathered from their files in the medical records department of the hospital and their theatre and ward records. Patient's biodata, etiology or mode of injury, part of body injured, type of injury sustained, places the injuries took place, the time between injury and presentation, pre-hospital care, complications of previous treatment, treatments received in our facility, etc., were collated and entered in a proforma designed for the purpose. Patients whose records were incomplete or whose case notes could not be located were excluded from the study.

Data were analyzed using IBM SPSS Statistics, version 21.0. (IMB, Inc., Armonk, US) and presented in the form of tables, charts, ratios, frequencies, and percentages. Mean, mode, median, and standard deviation were used for quantitative variables. Frequencies and percentages were used for qualitative variables.

## Results

A total of 90 children aged 0-16 years were included in this study, made of 58 boys (64.4%) and 32 girls (35.6%), with a male:female ratio of about 1.8:1.

**Figure 1:**
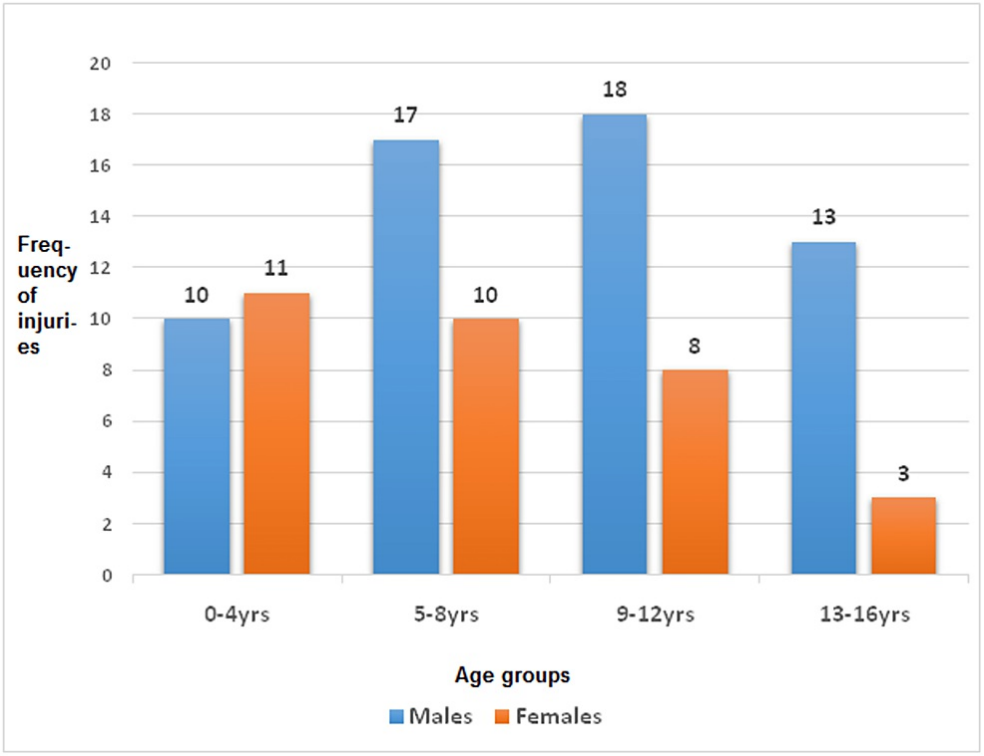
Frequency of injuries by age and sex

The mean age of all the children was 8.15+/-4.03 years. The youngest patient was a four-month-old boy who fell from his caregiver's arms and sustained a closed femoral shaft fracture. The median age was eight years, and the modal age was nine years. The average age of the boys (8.65+/-4.03) was a bit higher than that of the girls (7.25+/-4.01). Figure 1 shows the frequency of injuries in various age groups and in male and female children.

**Figure 2:**
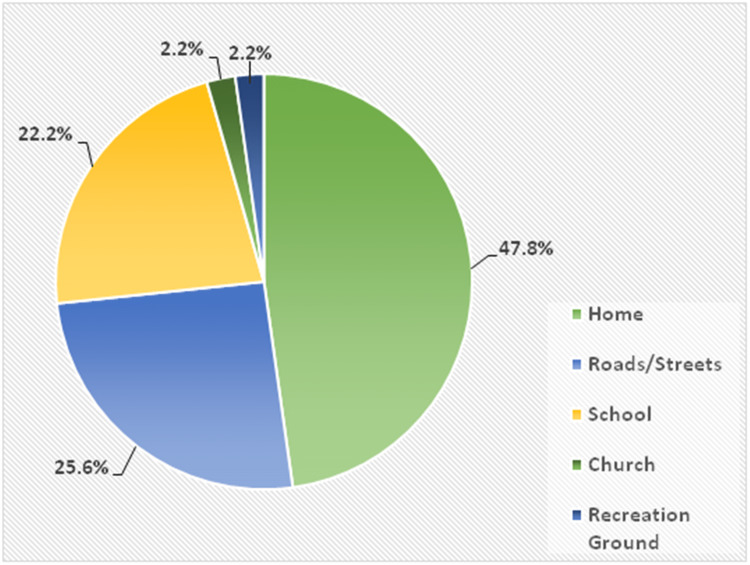
Places where injuries took place

The commonest place where injuries took place was the home in 43 patients (47.8%), as seen in Figure 2.

In terms of etiology or mode of injury, more than half of the patients (52, 57.8%) were injured as a result of a fall either at floor level or from a height (Table 1).

**Table 1:** Etiology of injuries

Etiology	Frequencies	Percentages (%)
Falls	52	57.8
Road traffic accidents (96% pedestrian)	21	23.3
Sports Injury	12	13.3
Pathological fractures	3	3.3
Assault/fight	2	2.2
Total	90	~100.0

The 90 patients studied sustained 96 musculoskeletal injuries, out of which 92 (95.8%) were close injuries and the remaining four (4.2%) were open. Eighty-four patients sustained one injury each, four patients sustained two injuries each, and one patient sustained three different injuries. Eighty-six patients (95.6%) sustained bony injuries, while the remaining four (4.4%) had only soft tissue injuries. Forty-nine injuries (51%) affected the upper limbs, while the remaining 47 (49%) injuries affected the lower limbs. The left side of the body was affected in 70.8% of cases, while the right side was affected in the remaining 29.2%.

The most frequently injured part of the body was the thigh in 37 cases (38.5%) in the lower limbs, followed by the elbow in the upper limbs in 25 cases (26.0%), as seen in Table 2.

**Table 2:** Parts of the body injured

Parts of the body	Frequencies	Percentages (%)
Pectoral girdle/shoulder	5	5.2
Arm	3	3.1
Elbow	25	26.1
Forearm	12	12.5
Wrist	4	4.2
Total upper/pectoral girdle	49	~51.0
Pelvis/acetabulum	2	2.2
Thigh	37	38.5
Leg	8	8.3
Total lower/pelvic girdle	47	49.0
Grand total (upper + lower limb)	96	~100.0

A total of 101 individual bones were fractured. Fifty-two (51.5%) of the 101 fractures occurred in the upper limbs, while the remaining 49 (48.5%) occurred in the lower limbs. The femoral shaft was the most frequently fractured bone (Table 3).

**Table 3:** Distribution of fractures in the bones of the limbs

Bones	Frequencies	Percentages (%)
Clavicles	3	3.0
Humerus	30	29.7
Radius	12	11.9
Ulna	7	6.9
Total upper limb fractures	52	51.5
Pelvis	2	2.0
Femur	36	35.6
Tibia	7	6.9
Fibula	4	4.0
Total lower limb fractures	49	48.5
Grand total number of fractures (upper + lower limbs)	101	100.0

Ninety-six (95.0%) of the fractures were complete fractures, two (2%) were green stick/buckle fractures, and three (3%) were epiphyseal injuries.

There were two joint dislocations, both of which presented as neglected unreduced injuries. One was an elbow dislocation in a nine-year-old boy with unreduced posterior elbow dislocation, and the other one was in an eight-year-old boy with sickle cell anemia who had an unreduced left hip dislocation and ipsilateral femoral shaft fracture from chronic osteomyelitis. Both had been previously managed by traditional bone setters (TBS).

Seventy-seven patients (85.6%) presented within hours or a few days of injury with their injuries being relatively fresh, while 13 (14.4%) patients presented, with various complications, to our center months (eight patients) and even years (five patients) after injury. The complications included nine malunions, two neglected joint dislocations, one infected nonunion of the humeral shaft, and one Volkmann's ischemic contracture of the forearm, wrist, and fingers from mismanaged forearm injury by TBS.

Of the 90 patients, 38 (42.2%) came directly from the scene of the accident to our hospital without previous treatment, 29 (32.2%) came after initial treatment in other hospitals, while the remaining 23 patients (25.6%) received treatment from TBS for variable periods before presenting to us. Treatments offered are as shown in Table 4.

**Table 4:** Treatments offered to patients ORIF - open reduction and internal fixation; COM - chronic osteomyelitis

Treatment	Frequencies	Percentages (%)
Open/closed reduction + K-wire fixation	21	19.0
Screws only fixation (femoral neck)	4	3.6
ORIF with plate and screws	6	5.5
Corrective osteotomies and osteoclasis for malunions	9	8.2
Wound debridement	7	6.4
Open reduction of neglected dislocations	2	1.8
External fixator applications	4	3.6
Closed reduction and cast applications	21	19.1
Skin traction	20	18.2
Intramedullary nailing	2	1.8
Wound care	4	3.6
Flap coverage	1	0.9
Bed rest - pelvic fracture	2	1.8
Partial thickness skin graft	2	1.8
Sequestrectomies for infected fractures/COM	2	1.8
Collar/cuff sling + figure of eight bandages (clavicular fractures)	2	1.8
Left against medical advice	1	0.9
Total	110	~100.0

Sixty-five patients had varying lengths of admission. Most of the patients (66, 73%) had good functional outcomes. Fifteen had joint stiffness that was managed with physiotherapy with good results. Significant malunion that required further treatment was seen in two patients, while superficial wound infections that responded to antibiotics were observed in seven patients. Two patients who presented with infected open fractures and osteomyelitis with pathological fractures ab initio had persistent residual bone infections at the last follow-up. Five patients had limb length discrepancies, one of which was significant and required a limb-lengthening procedure. One patient left against medical advice without receiving treatment.

## Discussion

Children are often vulnerable to injuries regardless of location for reasons that may include poor risk perception, living in environments adapted for adults, and inherent vulnerability. Most of these injuries are not reported [12].

Injuries are a major cause of morbidity and mortality in children and constitute a major part of the global disease burden. Many children who survive major trauma may be left with residual disabilities that could affect their education, development, and social life [2,16].

A total of 90 children were included in this study, out of which 58 (64.4%) were males and the remaining 32 (35.6%) were females, presenting a male:female ratio of 1.8:1. The preponderance of male children as the most frequently injured sex has been the usually observed trend in the literature [2,3,5,7,11,12,17,18]. The observed male:female ratio of 1.8:1 in this study is a bit lower than the ratio of 2.1:1 reported by Hussain et al. [5] and Verma et al. [17] but higher than the 1.6:1 and 1.5:1 ratio reported by Nesje et al. [2] and Hedström et al. [19], respectively. The reason for the preponderance of male children being injured may probably be related to genetic and hormonal factors that make them more active, with a tendency to take more risks during play and other activities. Hedström et al. [19] opined that the male preponderance is because boys are more likely to participate in sports and also take more risks, while Sural and Verma [12] said this observation is likely due to the fact that boys are more active, while girls are more likely to play sedentary games.

The average age of the children in this study was 8.15 years for both sexes combined, with a lower average of 7.25 years observed in girls only and a higher one of 8.65 years observed in boys. The combined mean of 8.15 years found in this study is similar to the mean age of 8.78 years reported by Omidiji et al. [20] in a study on fractures in children from Western Nigeria but much lower than the mean of 14 years observed by Sananta et al. [3] in a study from Indonesia. The latter authors, however, studied children between 0 and 17 years as opposed to between 0 and 16 years in this study.

The age group most frequently injured in this study for both sexes combined was the 5-12 years group (59.5%). Boys in this study were also most frequently injured in the same group, with 63.3% of injuries. However, ages 0-8 years were more affected in girls (67.7% of injuries in girls). The reason for this observed difference in peak ages of injury between both sexes is not clear and may need further investigation. Furthermore, peak age groups for musculoskeletal injuries in children of 6-12 years and 7-12 years for both sexes have been similarly reported by Song et al. [21] from China and Hussain et al. [5] from India, respectively. In contrast, higher peak periods of 12-18 years and 10 -17 years have been reported by Sananta et al. [3] and Nesje et al. [2] from Indonesia and Norway, respectively. In addition, the modal age of injuries in children of nine years seen in this study is lower than the 16 years observed by Sananta et al. [3]. The observed variations in the peak age of incidence of pediatric injuries may be due to environmental factors, including government policies on infrastructure, child protection policies, and the etiological factors leading to injuries.

The most frequent mode or etiology of musculoskeletal injuries in children in the literature appears to swing between falls and traffic accidents, with falls being observed as the commonest in most of the studies. Fall was the most frequently observed etiology in this study (58%), followed by traffic accidents (23%). Similarly, Singh et al. [7] and Hussain et al. [5], both from India, Kandel et al. [11] from Nepal, Hedström et al. [19] from Sweden, and Omidiji et al. [20] from Nigeria reported falls as the most frequent etiology for musculoskeletal injuries in pediatric patients in their studies. In contrast, both Sananta et al. [3] from Indonesia and Nesje et al. [2] from Norway reported traffic accidents as the most frequent cause of musculoskeletal injuries in children. These observed differences may be due to local childcare practices, traffic control policies, and enforcement of road signs, including the availability of zebra crossing points, separate tracks for children riding bikes, sidewalks or separate lanes for those walking on the roads, and other such safety measures.

In our study, the home (48%) was the commonest place where injuries took place in children. This is similar to the observations by some authors [5,12]. However, some other authors [2,3] reported that the street was the most common place where pediatric injuries took place. The findings by the last two authors are expected as they also reported traffic accidents as the most frequent cause of injuries in children in their studies. On a general note, the reason for more injuries occurring at home, as also reported in this study, may be explained partially by the fact that the growing child spends more time at home, especially in their early years of life.

In this study, upper limbs were observed to only be marginally (51%) more frequently injured than lower limbs. Hussain et al. [5], Singh et al. [7], and Kandel et al. [11] also found more upper limb injuries in their studies. In contrast, a marginal preponderance of lower limb injuries (51.87%) has been reported by Verma and Mahendra [17] in their study.

The side of the body (right or left) most frequently injured in children also appears to vary from study to study. Our study reported 70.1% of the injuries in children affected the left side of the body. Hussain et al. [5] also reported a similar observation. The reverse was observed in the studies by Singh et al. [7] and Kandel et al. [11], where right-side injuries predominated.

One hundred and one individual bones were fractured in our study, and the most frequently fractured bone was the femur (35.6%), followed by the humerus (29.7%). The femoral shaft was fractured in most cases (88.9%), while most humeral fractures (75.35) were supracondylar fractures. Femoral shaft fractures have similarly been reported as the most frequent fracture in children by Sural et al. [12]. This contrasts with the findings by several other authors who have reported the forearm bones as the most frequently fractured bones in children [3,5,19,22,23]. Kandel et al. [11], however, reported the humerus as the most frequent fracture bone in their series. Again, sociocultural practices, including participation in sports, childcare practices, housing, traffic control policies, and other environmental factors, may be contributory to the observed variations in findings by various authors.

In Nigeria, patients' presentation to appropriate hospitals is often delayed because patients first seek treatment most times from TBS [24-26]. This often leads to late presentations, and in many cases, patients present to the hospital for the first time with complications from the previous treatments, resulting in poorer outcomes of treatment. In this study, over 25% of patients presented to the hospital after delays ranging from weeks to years as a result of initial treatment by TBS. Similar intervention by quacks that leads to delays in hospital presentation and complications has also been reported by Hussain et al. [5] in a study from India. The story is, however, different in some other countries where patients tend to present quite early to the hospital for treatment, as reported by Sananta et al. [3] from Indonesia, which ensures better outcomes and reduced complications. There is a need for public enlightenment and advocacy in our country to encourage parents and guardians to take their injured children to hospitals as soon as possible instead of patronizing TBS and other quacks.

## Conclusions

In this study, falls and pedestrian traffic accidents were responsible for the majority of the injuries in children. Ensuring that children are supervised all the time, both at home and at school, will go a long in reducing injuries. Environmental hazards, such as wet floors that could lead to the child falling, should be avoided, in addition to not allowing children to climb on tables and chairs while playing. Designing houses with protective railings on balconies and staircases will also prevent falls from heights. To reduce traffic injuries, our roads and streets should be designed such that there is the provision of separate walkways, including separate biking tracks, as well as overhead bridges and zebra crossing points at regular intervals. Children below certain ages should be prevented from walking in the streets without an accompanying adult. Enlightenment of drivers to be more careful on busy streets with policies on speed limits in certain areas may also be necessary.

In addition, there is a need to establish more specialized trauma centers across our country to ensure easy access to appropriate specialists. Making treatment free for children of certain ages will also help to improve outcomes, as poverty is one of the reasons for delays in presentation as well as the patronage of TBS and other quacks, which further complicate the initial injuries.

## References

[1] Haagsma JA, Graetz N, Bolliger I (2016). The global burden of injury: incidence, mortality, disabilty adjustmentlife years and and time trends from the global burden of disease study 2013. Inj Prev.

[2] Nesje E, Valøy NN, Krüger AJ, Uleberg O (2019). Epidemiology of paediatric trauma in Norway: a single-trauma centre observational study. Int J Emerg Med.

[3] Sananta P, Julana R, Siahsaan LD (2020). Profile of patients with musculoskeletal injury in children at a tertiary referral hospital in Indonesia. Open Access Maced J Med Sci.

[4] World Health Organization (2009). Global health risks: mortality and burden of disease attributable to selected major risks. https://apps.who.int/iris/handle/10665/44203.

[5] Hussain S, Dar T, Beigh AQ, Dhar S, Ahad H, Hussain I, Ahmad S (2015). Pattern and epidemiology of pediatric musculoskeletal injuries in Kashmir valley, a retrospective single-center study of 1467 patients. J Pediatr Orthop B.

[6] Jespersen E, Holst R, Franz C, Rexen CT, Wedderkopp N (2014). Seasonal variation in musculoskeletal extremity injuries in school children aged 6-12 followed prospectively over 2.5 years: a cohort study. BMJ Open.

[7] Singh O, Gupta S, Din Darokhan MA, Ahmad S, Charak SS, Sen A (2018). Epidemiology of pediatric musculoskeletal injuries and their pattern in a tertiary care center of North India. Indian J Orthop.

[8] Odatuwa-Omagbemi DO, Inikori AK, Otene CI, Enemudo RET (2013). Musculoskeletal injuries: a cross-sectional study in a suburban teaching hospital. Niger J Orthop Trauma.

[9] Sathya C, Alali AS, Wales DC (2015). Mortality among injured children treated at different trauma center types. JAMA surg.

[10] Bickler SW, Rode H (2002). Surgical services for children in developing countries. Bull World Health Organ.

[11] Kandel IS, Acharya K, Gupta S, Shrestha B (2018). Spectrum of paediatric orthopaedic injuries in patients attending emergency department of Gandaki medical college of Pokhara, Nepal. MJPAHS.

[12] Sural S, Verma A (2015). The clinical profile of musculoskeletal injuries in children attending a major hospital in Delhi, India. J Clin Orthop Trauma.

[13] Grover JI, Ogirima MO (2010). Musculoskeletal Trauma. Paediatric Surgery: A Comprehensive Text For Africa.

[14] O’Connor S, Whyte E, Cheilleachair NN (2021). Sports and recreation musculoskeletal injuries in Irish primary school children. J Hum Sport Exerc.

[15] Cassel M, Müller J, Moser O, Strempler ME, Reso J, Mayer F (2019). Orthopedic injury profiles in adolescent elite athletes: a retrospective analysis from a sports medicine department. Front Physiol.

[16] World Health Organization (2008). World report on child injury prevention. https://pubmed.ncbi.nlm.nih.gov/26269872/.

[17] Verma V, Mahendra M (2022). Epidemiology of pediatric musculoskeletal trauma patients admitted to the Trauma Center of King George's Medical University (KGMU) during COVID-19-induced lockdown. Cureus.

[18] Ibrahim S, Regis AAJ, Leopold KK (2022). Severe musculoskeletal injuries in children during play. Open J Orthop.

[19] Hedström EM, Svensson O, Bergström U, Michno P (2010). Epidemiology of fractures in children and adolescents. Acta Orthop.

[20] Omidiji OTA, Akinmokun OI, Olowoyeye OA (2022). Epidemiological and radiological patterns of paediatric fractures in an elite community in south-western Nigeria. J Clin Sci.

[21] Song F, Zeng Y, Tian J (2020). Epidemiology and the economic burden of paediatric fractures in China: A retrospective study of 14,141 fractures. Bone.

[22] Wolfe JA, Wolfe H, Banaag A, Tintle S, Perez Koehlmoos T (2019). Early pediatric fractures in a universally insured population within the United States. BMC Pediatr.

[23] Baig MN (2017). A review of epidemiological distribution of different types of fractures in paediatric age. Cureus.

[24] Odatuwa-Omagbemi DO, Enemudo RET, Enamine SE, ESezobor EE (2014). Traditional bone setting in the Niger Delta region of Nigeria. Niger J Med.

[25] Alonge TO, Dongo AE, Notidge TE, Omololu AB, Ogunlade SO (2004). Traditional bone setters in South-Western Nigeria: friends or foes. West Afr J Med.

[26] Odatuwa-Omagbemi DO (2012). Complications of traditional bone setters practice in Nigeria: need for urgent action. Niger J Clin Sci.

